# Diarrhea in young children from low-income countries leads to large-scale alterations in intestinal microbiota composition

**DOI:** 10.1186/gb-2014-15-6-r76

**Published:** 2014-06-27

**Authors:** Mihai Pop, Alan W Walker, Joseph Paulson, Brianna Lindsay, Martin Antonio, M Anowar Hossain, Joseph Oundo, Boubou Tamboura, Volker Mai, Irina Astrovskaya, Hector Corrada Bravo, Richard Rance, Mark Stares, Myron M Levine, Sandra Panchalingam, Karen Kotloff, Usman N Ikumapayi, Chinelo Ebruke, Mitchell Adeyemi, Dilruba Ahmed, Firoz Ahmed, Meer Taifur Alam, Ruhul Amin, Sabbir Siddiqui, John B Ochieng, Emmanuel Ouma, Jane Juma, Euince Mailu, Richard Omore, J Glenn Morris, Robert F Breiman, Debasish Saha, Julian Parkhill, James P Nataro, O Colin Stine

**Affiliations:** 1 University of Maryland, College Park, MD, USA; 2 Wellcome Trust Sanger Institute, Hinxton, Cambridgeshire, UK; 3 University of Maryland, School of Medicine, Baltimore, MD, USA; 4 Medical Research Council Unit, Serrekunda, Gambia; 5 International Centre for Diarrhoeal Disease Research, Bangladesh, Dhaka, Bangladesh; 6 Kenya Medical Research Institute (KEMRI)-US Centers for Disease Control and Prevention Research Collaboration, Kisumu, Kenya; 7 Center for Vaccine Development, Bamako, Mali; 8 University of Florida, Gainesville, FL, USA; 9 Emory University, Atlanta, Georgia, USA; 10 University of Virginia, Charlottesville, VA, USA

## Abstract

**Background:**

Diarrheal diseases continue to contribute significantly to morbidity and mortality in infants and young children in developing countries. There is an urgent need to better understand the contributions of novel, potentially uncultured, diarrheal pathogens to severe diarrheal disease, as well as distortions in normal gut microbiota composition that might facilitate severe disease.

**Results:**

We use high throughput 16S rRNA gene sequencing to compare fecal microbiota composition in children under five years of age who have been diagnosed with moderate to severe diarrhea (MSD) with the microbiota from diarrhea-free controls. Our study includes 992 children from four low-income countries in West and East Africa, and Southeast Asia. Known pathogens, as well as bacteria currently not considered as important diarrhea-causing pathogens, are positively associated with MSD, and these include *Escherichia/Shigella*, and *Granulicatella* species, and *Streptococcus mitis/pneumoniae* groups. In both cases and controls, there tend to be distinct negative correlations between facultative anaerobic lineages and obligate anaerobic lineages. Overall genus-level microbiota composition exhibit a shift in controls from low to high levels of *Prevotella* and in MSD cases from high to low levels of *Escherichia/Shigella* in younger versus older children; however, there was significant variation among many genera by both site and age.

**Conclusions:**

Our findings expand the current understanding of microbiota-associated diarrhea pathogenicity in young children from developing countries. Our findings are necessarily based on correlative analyses and must be further validated through epidemiological and molecular techniques.

## Background

Diarrheal diseases continue to be major causes of childhood mortality, ranking among the top four largest contributors to years of life lost in sub-Saharan Africa and South Asia [1]. The proportion of deaths attributed to diarrhea among children aged under 5 years is estimated to be approximately 15% worldwide [2], and as high as approximately 25% in Africa and 31% in South East Asia [3]. More than two dozen enteric pathogens, belonging to diverse branches of the tree of life, are known to cause diarrhea and can be tested for in a clinical setting. However, it is likely that additional pathogens remain to be identified among the enteric microbiota.

In response to important unanswered questions surrounding the burden and etiology of childhood diarrhea in developing countries, the William and Melinda Gates Foundation commissioned the Global Enterics Multicenter Study (GEMS) [4], which recently reported the pathogens responsible for cases of moderate-to-severe diarrhea (MSD) in seven impoverished countries of sub-Saharan Africa and south Asia. Importantly, for approximately 60% of MSD cases in GEMS, no known pathogen could be implicated by conventional diagnostic methods [5]. These observations highlight the potential presence of previously undiscovered pathogens, and/or possible interactions between pathogens and other members of the intestinal microbiota (both pathogenic and commensal) that may either exacerbate the clinical manifestation or protect the host from disease.

Here we apply molecular techniques to survey the intestinal microbiota in a subset of GEMS cases and controls. Our study comprises 992 children from four under-developed countries in West Africa (The Gambia and Mali), East Africa (Kenya), and South Asia (Bangladesh), representing a subset of the over 25,000 GEMS children enrolled. Our results shed additional light on potential mechanisms underlying MSD in children of developing countries. Prior to presenting these results we would like to stress that our analyses are, by necessity, correlative and the results presented here must be validated through epidemiological and molecular analyses, several of which are already underway.

## Results and discussion

### Description of data

Our data comprise roughly equal proportions of cases and controls (0.51 *vs.* 0.49, respectively) from four sites: Bangladesh (N = 206), The Gambia (N = 269), Kenya (N = 305), and Mali (N = 212). Approximately 55% of the subjects were boys. Of 992 samples, 508 were from patients with MSD (Table 1). The children ranged in age from newborn to 59 months. We stratified them into five age categories: 0 to 5 months (N = 112), 6 to 11 months (N = 308), 12 to 17 months (N = 173), 18 to 23 months (N = 146), and 24 to 59 months (N = 253). There were no significant differences between the proportion of cases and controls in each country and from each age group (Table 1). The sequencing of PCR amplified 16S rRNA genes resulted in 3,584,096 reads passing quality checks. Each sample had at least 1,000 reads, and there were an average of 3,613 reads per sample. The reads were clustered using DNAclust [6] into 97,666 operational taxonomic units (OTUs). Of these, 21,247 passed chimera checking, were detected in more than five samples, or represented at least 20 sequences in a single sample, and were included in further analysis. The number of OTUs per sample ranged from 55 to 1252, with a median of 380 and an average of 412. The mean OTU size was 138, ranging from 5 (by definition) to 192,978 (with median OTU size = 15 sequences). Representative sequences from the 21,247 OTUs matched 728 distinct taxa from 161 genera. Among these, 4,730 (22 %) did not have good (>100 bp exact match, >97% identity) matches to isolate sequences from the Ribosomal Database Project (RDP). These were flagged as ‘unassigned’ in our analysis and are discussed further below. These sequences are not simply an artifact of our stringent alignment criteria as evidenced by the fact that a re-analysis of the 6,879 most abundant OTUs using the reference-based OTU picking algorithm implemented in Qiime [7] failed to classify a similar proportion of sequences (2,162 or 31% of the abundant OTUs).

**Table 1:** Demographics of the children

**Demographic characteristics for samples (N = 992), N (%)**				
	**MSDN = 508**	**ControlsN = 484**	***P *** **value**	**TotalN = 992**
Age groups by months			0.1788	
0 to 5	58 (11)	54 (11)		112 (11)
6 to 11	171 (34)	137 (28)		308 (31)
12 to 17	93 (18)	80 (17)		173 (17)
18 to 23	70 (14)	76 (16)		146 (15)
24 to 59	116 (23)	137 (28)		253 (26)
Country			0.3622	
The Gambia	138 (27)	131 (27)		269 (27)
Mali	110 (22)	102 (21)		212 (21)
Kenya	165 (32)	140 (29)		305 (31)
Bangladesh	95 (19)	111(23)		206 (21)
Gender			0.5785	
Male	286 (56)	264 (54)		550 (55)
Female	222 (44)	220 (46)		442 (45)
Dysenteric stools				
	140 (28)	7 (1)	<10^-16^	147 (15)

All ages are in months. *P* values test independence of MSD cases and controls with regards to demographic variable. *P* values for age in months (treated as a continuous variable) computed by independent samples t-test. *P* values for categorical variables calculated using chi-square test.

MSD: Moderate-to-severe diarrhea.

### Microbiota variations by age

**Figure 1:**
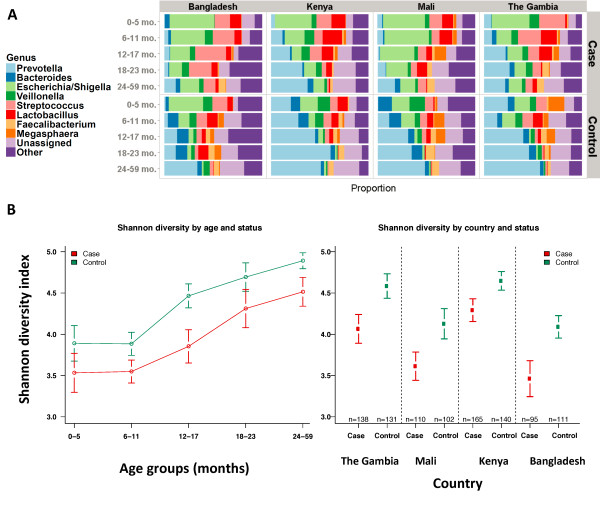
**Comparison of diarrheal and non-diarrheal stool. (A)** Proportional abundance of genera in non-diarrheal controls and MSD cases in different age categories. Each color represents a different group. The order and color for each group is the same for controls (patients without MSD) and MSD cases. The eight groups most frequently found in controls (*Prevotella, Bacteroides, Escherichia/Shigella, Veillonella, Streptococcus, Lactobacillus, Faecalibacterium, Megasphaera*, plus unassigned and other) are depicted. **(B)** Shannon diversity index across ages and diarrheal status. Average Shannon diversity indices for the five different age strata as well as the corresponding 95% confidence intervals. Both cases and controls exhibited higher mean Shannon diversity index scores at higher age groups compared to lower age groups (*P* <0.001, one-way ANOVA). The diversity of healthy samples positively correlates with age in the first 2 years of life, as previously reported [12]. The diversity index for cases is significantly less than that for controls within each country (*P* <0.02, Tukey’s t-test corrected for multiple comparisons). Also see Additional file 2: Table S2 and Additional file 3*:* Figure S1.

The well documented [8-10] succession of the intestinal microbiota during child development is apparent in our non-diarrheal control samples (Figure 1A). During the first year of life, the ‘healthy’ gut microbiota in our infant cohorts is characterized by comparatively low overall diversity and a relatively high proportion of facultatively anaerobic, and potentially pathogenic, organisms (for example, the *Escherichia/Shigella* group, which cannot be distinguished from each other by 16S rRNA gene sequences), organisms that are believed to play a role in the development of the host immune system [11,12]. In older ages, the dominance of these organisms is reduced, replaced by a corresponding increase in overall diversity (Figure 1B), accompanied by a particularly pronounced increase in the proportional abundance of the bacterial genus *Prevotella*. These changes are most evident in our non-diarrheal control samples, where the genus *Prevotella* increases from approximately 12% to approximately 48% proportional abundance during the first 5 years of life, while the *Escherichia* genus drops from about 20% proportional abundance in infants under 6 months of age to approximately 1% in 2- to 5-year-olds (Additional file 1: Table S1). Two other genera, *Veillonella* and *Streptococcus* also exhibit significant decreases with increasing age. Our data also show an increase with increasing age in the proportion of a range of organisms (labeled ‘unassigned’ in Figure 1A and Additional file 1: Table S1) that have no good quality matches to cultured isolates in public databases, and which appear to belong predominantly to obligate anaerobic bacteria (over 60% can be assigned by the RDP classifier to the *Ruminococcaceae* and *Lachnospiraceae* families of the Firmicutes phylum, which are relatively poorly represented in culture collections [13], as well as the *Bacteroidaceae* family). These previously-uncultured putative obligate anaerobes increase in proportional abundance from approximately 8% in diarrhea-free young children to approximately 23% in the older age group, consistent with increase in diversity within the intestinal microbiota and the known expansion of these groups, which are able to colonize the intestine in greater numbers as the complex polysaccharides they utilize for growth become a greater feature of the host diet [14].

These observations broadly hold when stratifying by country of origin; however, country-specific effects are also apparent. For example, the samples from Bangladesh are different from the African countries, particularly in the younger age groups, and are characterized by a lower proportion of *Prevotella* sequences and a higher proportion of organisms from the *Escherichia/Shigella* and *Streptococcus* genera (Figure 1A and Additional file 1: Table S1).

The patterns observed within control samples were significantly different from patterns from patients with MSD; however, some overall age-related trends were similar. For example, *Prevotella* abundance correlates with age, albeit reaching a much lower peak, with only 23% abundance in the oldest age group (*vs.* 48% in controls, *P* <10^**-**16^). Other obligate anaerobic microbes have lower proportional abundance among cases compared to controls: *Bacteroides* and the unclassified putative anaerobes are both 5% lower in cases, consistent with previous observations that indicate intestinal dysbiosis is associated with a decrease in the proportional abundance of obligate anaerobes [15]. Among cases, *Escherichia/Shigella* and *Streptococcus* spp. maintain a high proportion across all age groups, though their preponderance drops significantly (41% to 13% and 18.5% to 7.5%, respectively) as children age. Furthermore it appears that *Prevotella* and *Escherichia/Shigella* are negatively correlated in MSD cases (Spearman rho = -0.55, *P* <0.0001). The disruption associated with diarrhea is also reflected in lower diversity values in MSD cases in every age group (Figure 1B, Additional file 2: Table S2, Additional file 3: Figures S1A-D).

Country-specific effects were also observed in diarrheal stool; for instance, in Kenya, diarrhea appeared to have a less marked effect on the microbiota (Figure 1A and Additional file 1: Table S1). *Escherichia/Shigella* spp. were most common in Mali, accounting for 34% of the sequences, next most common in Bangladesh (24%) and least common in The Gambia (15%). *Prevotella* spp. were found in high proportional abundances in The Gambia (18%) and Kenya (19%). The genus *Streptococcus* is found in relatively high abundances in Bangladesh (21%) and The Gambia (13%) with lower abundances in Mali (10%) and Kenya (9%). As expected, the taxonomic diversity (Shannon diversity index) is significantly different between cases and controls in all countries (*P* <0.005, pairwise t-test). Of note, where *Prevotella* is more common (The Gambia and Kenya), the diversity is higher (Figure 1B).

### Taxonomic groups statistically increased or decreased in diarrhea

Multidimensional scaling analysis could not separate the diarrhea and diarrhea-free bacterial communities due to high inter-personal variation (Additional file 3: Figure S3). We estimated the association of individual OTUs with disease using statistical tests addressing both presence-absence statistics (Fisher’s exact test and logistic regression) and abundance-dependent statistics (using generalized linear models) that account for the number of OTU-specific sequences in each stool, and potential confounders such as sampling depth, age, and country (see Additional file 4: Table S3 for a full summary). The former address similar questions to those commonly targeted by the traditional culture-based epidemiological studies, while the latter allow us to assess how pathogen proportional abundance correlates with morbidity.

Ten OTUs were found to be positively associated with diarrhea by all statistical tests. The OTUs associated with MSD have high-similarity matches against database sequences from bacterial taxa in the *Escherichia/Shigella*, *Granulicatella* spp*.*, and *Streptococcus mitis/pneumoniae* groups. When only abundance-dependent statistics are used to determine significance, an additional 18 OTUs are found to be highly associated with diarrhea, corresponding to the bacterial species *Escherichia/Shigella*, *Campylobacter jejuni,* and *Streptococcus pasteurianus*. When only considering presence/absence statistics, 43 additional OTUs are found to be associated with diarrhea, comprising the bacterial groups already discussed above as well as members of the genera *Lactobacillus*, *Neisseria*, *Citrobacter*, *Erwinia,* and *Haemophilus*. It is noteworthy that all of these organisms are either facultatively anaerobic or microaerophilic.

On the other hand, there were no OTUs positively associated with healthy stools by both statistical methods, reflecting the higher degree of inter-individual variation in microbiota content in healthy individuals. Considering only presence/absence statistics, there are 43 OTUs associated with non-diarrheal control samples. The genera associated with these control samples include members of the clostridial families *Peptostreptococcaceae*, *Eubacteriaceae,* and *Erysipelotrichaceae*, and the genera *Clostridium sensu stricto*, *Dialister*, *Enterococcus*, *Prevotella*, *Ruminococcus,* and *Turicibacter*. When considering only abundance statistics, an additional 19 OTUs are significantly associated with non-diarrhea samples and have high quality matches to database sequences corresponding to *Bacteroides fragilis*, *Dialister*, *Megasphaera*, *Mitsuokella/Selenomonas*, *Prevotella* spp., and *Clostridium difficile*. Thus, it can be seen that many obligate anaerobic bacterial lineages correlate with healthy status.

### Functional differences between cases and controls

The broad statements made above about oxygen tolerance in the diseased microbiota are supported by PICRUST [16] analyses of our data. Specifically, this showed putative signatures of obligate anaerobic gut lineages to be enriched in the diarrhea-free samples (for example, glycolysis, *P* = 10^-9^; pyruvate metabolism, *P* = 10^-7^; short chain fatty acid biosynthesis, *P* = 10^-3^; xylene degradation, *P* = 10^-7^; and so on; all *P* values by Welch’s t-test as computed by STAMP [17]), while oxygen dependent pathways (for example, the TCA cycle, *P* <10^-15^) are enriched in diseased samples.

### Taxonomic groups correlated with dysentery

**Figure 2:**
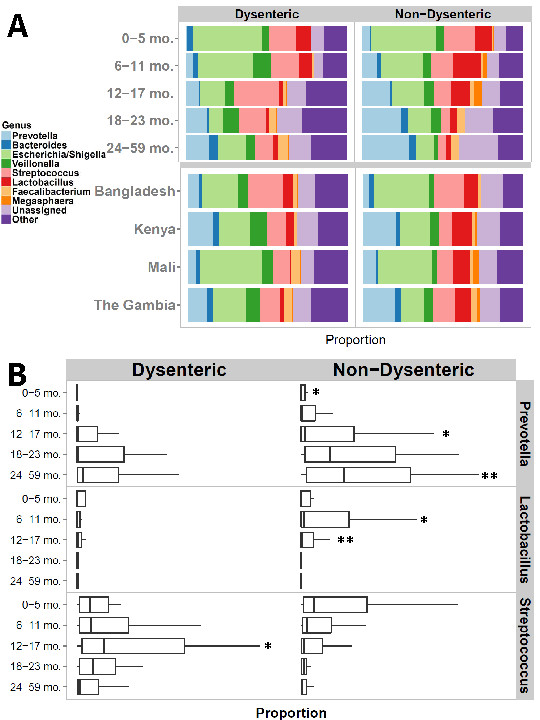
**Comparison of dysenteric and non-dysenteric stool. (A)** Genus-level comparison of dysenteric and non-dysenteric diarrheal stool (top) stratified by age; (bottom) stratified by country. **(B)** Proportional abundance boxplots of *Prevotella, Lactobacillus*, and *Streptococcus* in dysenteric and non-dysenteric diarrheal stools by age category. The upper whisker extends from the 75th percentile to the highest value that is within 1.5 * IQR of the hinge, where IQR is the inter-quartile range, or distance between the first and third quartiles. The lower whisker extends from the hinge to the lowest value within 1.5 * IQR of the hinge. Data beyond the end of the whiskers are outliers and are not plotted. Asterisks above the whisker indicate a statistically significant difference (by t-test) between dysenteric and non-dysenteric stools placed in the panel with the more abundant mean. A single asterisk indicates *P* <0.05; double asterisks indicate *P* <0.01. *Prevotella* is significantly associated with non-dysenteric cases overall (*P* = 0.0003) and in age groups 0 to 6 months (*P* = 0.01), 12 to 17 months (*P* = 0.03), and 24 to 59 months (*P* = 0.001). *Lactobacillus* is significantly associated with non-dysenteric cases overall (*P* = 0.0002) and in children 6 to 11 months (0.02) and 12 to 17 months (*P* = 0.003), while the genus *Streptococcus* is associated with dysentery overall (*P* = 0.007), particularly in children aged 12 to 17 months (*P* = 0.01).

We segregated diarrheal stool based on diagnosis of dysentery (presence of blood) and found a total of 30 OTUs that were strongly correlated with dysentery when comparing with non-dysentery diarrheal stool (metagenomeSeq [18], *P* <0.05). These include several well-known pathogens such as *Enterococcus faecalis*, *Campylobacter jejuni*, *Bacteroides fragilis*, *Clostridium perfringens*, *Enterobacter cancerogenus*, and members of the *Granulicatella*, *Haemophilus*, *Klebsiella,* and *Escherichia/Shigella* genera. Also associated with dysentery were members of the *Streptococcus pasteurianus* and *Streptococcus salivarius* groups. A single OTU, corresponding to *Lactobacillus ruminis*, was found to be negatively associated with dysentery. A genus-level representation of these findings is shown in Figure 2.

### Network view of diarrheal illness

The overall results presented above are also borne out in correlation networks constructed from the data (Additional file 3: Figure S7). At the broad level, in both MSD cases and controls, it can be seen that there tend to be negative correlations between facultative anaerobic lineages and obligate anaerobic lineages. The most obvious example is the negative correlation of the potentially protective *Prevotella* genus with that of potential pathogens such as *Escherichia/Shigella*. Similarly, there are also positive correlations within these two phenotypic subgroupings, such that obligate anaerobic genera such as *Prevotella*, *Roseburia,* and *Dialister* are correlated with each other, while facultative anaerobic or microaerophilic genera such as *Streptococcus*, *Lactobacillus*, *Escherichia/Shigella*, and other Proteobacteria are also correlated with each other. The diarrhea-free network appears to be more tightly connected than the diarrheal network, consistent with ecological theories that equate environment diversity and connectedness with ecosystem stability/health [19,20]. At the same time, we would like to note that our data do not allow a reliable quantitative assessment of such phenomena due to the large level of inter-personal variation.

## Discussion

Our analysis of the 16S rRNA gene-based taxonomic profile of diarrheal and control stool samples has demonstrated a strong association between acute diarrheal disease and the overall taxonomic composition of the stool microbiota in young children from the developing world. We have identified statistically significant disease associations with several organisms already implicated in diarrheal disease, such as members of the *Escherichia/Shigella* genus and *C. jejuni*. In addition, we have uncovered an association with diarrheal disease for several organisms not widely believed to cause this disease, such as *Streptococcus* and *Granulicatella*. Streptococcal OTUs associated with disease primarily belong to either the *Streptococcus pneumoniae/mitis* group (indistinguishable within the 16S rRNA gene regions targeted by our study), which contains several important human pathogens, or the *Streptococcus pasteurianus* group. These results merit further exploration as recent studies provide evidence of *Streptococcus*-related diarrheal cases [21,22]. It is important to stress that pathogenicity is only one of many possible explanations for these findings and the organisms associated with disease status may also either: (1) usually inhabit the upper GI tract and become apparent in diarrheal stool due to dislodging and reduced transit time during disease; (2) thrive in disturbed gut environments; (3) may be better able to persist/resist dislodgement during a diarrheal purge; or (4) a combination of pathogens may cause disease in these children [23]. Prior evidence certainly suggests that facultative anaerobes (many of which we find associated with diarrhea) tend to flourish in a variety of perturbed gut environments, possibly because the reducing power of the microbiota is affected by the loss of obligate anaerobes following perturbation [15]. Any causality would need to be demonstrated through further experimentation. At the same time, streptococci are also found in our study to be associated with more severe forms of diarrhea (dysentery), thereby strengthening the case for a possible causal connection. Despite uncertainty regarding the causes and effects of microbiota perturbations in the setting of MSD, dissecting the physiologic implications is warranted. For example, an increase in streptococcal or other species in the setting of diarrhea may confer or exacerbate diarrheal effects. *S. mutans* has recently been postulated to have a role in human enteritis. Our work represents an important first step in understanding the complex interaction between microbiota and diarrheal pathogens in developing country settings.

Our study has also revealed a high prevalence of members of the *Prevotella* genus (primarily *Prevotella copri*) in the stool of developing world children, as well as the negative correlation of this genus with disease. These organisms are prevalent in the developing world [14], yet are relatively poorly studied due to fairly low prevalence in the industrialized world [24]. Samples containing high proportions of members of the *Prevotella* genus also have higher overall bacterial diversity, potentially driven by the level of complex polysaccharides/starchy fiber in the diet. Recent evidence suggests that *Prevotella* spp. are particularly abundant in rural African children consuming a high fiber diet [25]. This is in stark contrast to Western children, who typically have much higher abundances of *Bacteroides* spp., and very little *Prevotella*, a difference that is believed to be linked to diet [26].

Our co-occurrence network analyses (Additional file 3: Figure S7) and proportional abundance analysis (Additional file 1: Table S1) suggest potential negative interactions between *Prevotella* and enteric pathogens, such as members of the *Escherichia/Shigella* genus, raising the possibility for the development of novel *Prevotella*-based therapeutic strategies. Another possible probiotic organism identified in our study is *Lactobacillus ruminis*. This organism was found to be associated with non-diarrheal stool and also with less severe forms of diarrhea when comparing diarrheal to dysenteric stool. Although the increase in frequency of these taxa in diarrhea could be due to shortened intestinal transit time, the difference in prevalence of *Lactobacillus* between cases of MSD and dysentery are less likely to represent this effect. *Lactobacillus ruminis* has immunomodulatory properties and has been previously suggested as a potential probiotic [27].

Among OTUs found associated with non-diarrheal stool are sequences classified as *Clostridum difficile*, a surprising finding given that this organism is a common cause of enteric disease, primarily in hospitalized elderly patients. However, although *C. difficile* can be an important pathogen, it is actually carried asymptomatically by around 60% of infants [28]. We also found a conflicting association of OTUs assigned as *Bacteroides fragilis* with both the diarrhea-free status and dysentery, a finding that can perhaps be explained by strain-to-strain variation. Enterotoxigenic *B. fragilis* strains are well characterized diarrheal agents in children [29] whereas, in contrast, non-toxigenic *B. fragilis* has been linked to anti-inflammatory protective effects in mouse models [30]. It is therefore possible that different strains, which cannot be differentiated through 16S rRNA gene sequencing, might account for these opposing results.

Our study identified many sequences that do not have good matches against cultured organisms in current 16S rRNA gene databases. Many of these sequences only have high-quality matches to other uncultivated and uncharacterized intestinal microbes, highlighting the presence of a large reservoir of uncharacterized microbes in the intestinal tract of children within the developing world, as reported before [31]. Many of the unknown sequences appear to belong to obligate anaerobic lineages of the Firmicutes phylum, which are under-represented in culture collections compared to other intestinal dwelling groups such as *Bacteroides* and bifidobacteria. The prevalence of such ‘unknown’ sequences is higher in controls and several of these uncharacterized organisms exhibit strong associations with diarrhea-free samples, highlighting their potential role in the maintenance of a healthy gut microbiota, and suggesting the need for a better in-depth characterization of the gut microbiota of children within the developing world, complementing resources recently developed in Europe [32] and the US [33].

Our observations related to the microbial succession in the developing infant gut microbiota carry several caveats. A single sample was collected from each child at a single point in time, and we lack extensive data on prior history of diarrhea. While the data are suggestive of a progression in microbiota structure, monitoring of a birth cohort will be necessary to fully understand the progression of gut microbiota, and assess the impact of diarrhea (including, potentially, multiple episodes of diarrhea) on this process. At a technical level, we would also note that the primer sets used in this study (targeting the V1-V2 hypervariable regions of the 16S rRNA gene) do not effectively amplify bifidobacteria [34,35], known to be dominant members of the intestinal microbiota of breast-fed infants, but this bias is likely to be uniform between cases and controls. We purposefully selected a primer set better targeted towards bacterial groups containing known and potential pathogens, such as *Enterobacteriaceae*, to improve our chances of detecting novel pathogens at the cost of obtaining less information about the already well-established early dominance by bifidobacteria.

Our study revealed the limitations of existing molecular and bioinformatics approaches employed in a clinical setting for performing taxonomic surveys of stool samples. The use of the 16S rRNA gene, for example, does not afford a sufficient discrimination within taxonomic groups containing known or putative pathogens (*Escherichia/Shigella*, *Streptococcus*, and so on) indicating the pressing need for the development of new cost-effective and relatively unbiased molecular approaches [36] for increasing the resolution of epidemiological surveys such as ours. Relatedly, the accurate taxonomic assignment of sequences generated in studies such as ours is hampered by numerous errors in public databases and by the use of simplistic ‘lowest common ancestor’ heuristics by software tools faced with ambiguous taxonomic information. The results presented in this paper were obtained through the careful manual annotation of all the OTUs found to be associated with disease state (see Additional file 5: Table S4). Finally, we had to develop a novel statistical method [18] for identifying disease association in order to appropriately address data rarefaction as well as to control for the high inter-personal variability, a typical feature of the healthy gut microbiota [37], and other confounding factors.

## Conclusions

Overall our study demonstrates that the major differences in the microbiota between diarrheal and normal stools are quantitative differences in the proportions of the most prevalent taxa. Such quantitative differences were also observed in our previous qPCR-based study where we found that 80% (1,665/2,072) of controls and 89% (1,307/1,461) of MSD cases had detectable levels of *Shigella*. Quantitative measurements of *Shigella* abundance were critical to assessing attributable risk [38]. Among the known causes of diarrhea (rotavirus, *Shigella*, *Cryptosporidium*, Enterotoxigenic *E. coli*, and so on) the attributable fraction of diarrhea in young children is estimated to be just 43% [5]. Our study provides initial evidence for the existence of novel pathogenic agents. The most likely candidates from our study are members of the *Enterobacteriaceae* and streptococci, taxa which already contain many known human pathogens. Further exploration of these organisms is necessary to better understand their pathogenic potential and the likelihood of their emergence as major pathogens through the acquisition of additional pathogenicity factors. Importantly, our study reveals a possible protective role against diarrhea for the *Prevotella* genus and *Lactobacillus ruminis*. Understanding such effect is important. For example, microbiological [39] or dietary [26] interventions may be possible in the supportive treatment of diarrhea in children similar to approaches used in the management of enteric infections in adults [39-41]. Further genomic and epidemiological studies are necessary to better characterize this genus and to assess the potential development of diet- or microbiological-based therapeutics.

## Materials and methods

### **Study design and participants**

Stool samples were selected from a large case/control study of moderate-to-severe diarrhea in children aged under 5 years [42]. Cases were enrolled upon presentation to a health clinic reporting MSD. MSD eligibility criteria included sunken eyes, loss of normal skin turgor, a decision to initiate intravenous hydration or to hospitalize the child, or the presence of blood in the stool. Controls were sought following case enrollment, sampled from a demographic surveillance database of the area. Individuals were excluded if they were unable to produce a sufficient amount of stool volume for testing or they were unable or unwilling to consent to involvement in the study. Every participant was consented prior to collection of their stool and their data. Consent was given by the caregiver (usually mother) because the patients are all children aged less than 5 years. All samples were collected between March of 2008 and June of 2009. One sample was collected for each child and no time-series analyses were conducted. The Institutional Review Boards (IRBs) at all cooperating institutions have reviewed and approved the protocol. The IRB Federal Wide Assurance numbers for all the sites are as follows: University of Maryland Baltimore FWA00007145, The Gambia, Medical Research Council Labs FWA 00006873, Kenya Medical Research Institute FWA 00002066, University of Mali Faculty of Medicine Pharmacy and Dentistry FWA 00001769, and International Centre for Diarrhoeal Disease Research, Bangladesh FWA 00001468. Further details on study design are described by Kotloff *et al.*[42].

### **Microbiology methods**

Stool specimens were collected in sterile containers and examined within 24 h. Stools were stored at 2 to 8°C while in transit to the laboratory. Each fresh stool specimen was aliquoted into multiple tubes. All samples were analyzed by traditional microbiological tests for known bacterial, viral, and eukaryotic pathogens. Details of these methods can be found in Panchalingam *et al*. [43] DNA was isolated using a bead beater with 3 mm diameter solid glass beads (sigma Life Science), and subsequently 0.1 mm zirconium beads (BIO-SPEC Inc.) to disrupt cells. The cell slurry was then centrifuged at 16,000 *g* for 1 min, the supernatant removed and processed using the Qiagen QIAamp® DNA stool extraction kit. Extracted DNA was precipitated with 3 M sodium acetate and ethanol and the DNA shipped to the USA.

### Amplification and sequencing

DNA was amplified using ‘universal’ primers targeting the V1-V2 region of the 16S rRNA gene (small subunit of the ribosome) in bacteria (338R (5’- CATGCTGCCTCCCGTAGGAGT-3’ and 27 F (5’-AGAGTTTGATCCTGGCTCAG-3’). Both forward and reverse primers had a 5’ portion specific for use with 454 FLX sequencing technology and the forward primers contained a barcode between the FLX and gene specific region, so that samples could be pooled to a multiplex level of 96 samples per instrument run (see Additional file 6: Table S5 for barcode information).

### Data availability

Sequencing data and sample metadata are available at the NCBI archive under project PRJNA234437.

Source code and documentation for the analysis pipeline are available at GitHub: [44].

Abundance table and metadata are available, in BIOM [45] format, at [46].

Additional information on the study as well as links to all resources outlined above are made available at [47].

### Analysis pipeline

The individual reads were filtered for quality using custom in-house scripts that perform the following checks suggested in Huse *et al.*[48]: (1) sequences containing any ambiguity codes (N) are removed; (2) sequences that were shorter than 75 cycles of the 454 instrument were removed (each cycle yields an average of 2.5 bp depending on the sequence composition); (3) sequences for which a barcode could not be identified were removed. These checks are similar to those that can be performed by Mothur [49]. The high quality sequences were separated into 992 sample-specific sets according to the multiplexing barcodes. Conservative OTUs were clustered using DNAclust [6] with parameters (-r 1) (99% identity radius) thus ensuring that the definition of an OTU is consistent across all samples. To obtain taxonomic identification, a representative sequence from each OTU was aligned to Ribosomal Database (RDP) [50] (rdp.cme.msu.edu, release 10.4) using blastn with long word length (-W 100) in order to only detect nearly identical sequences. Sequences without a nearly identical match to RDP (>100 bp perfect match and >97% identity, as defined by BLAST) were marked as being ‘unassigned’ and assigned an OTU identifier. The resulting data were organized into a collection of tables at several taxonomic levels containing each taxonomic group as a row and each sample as a column.

We note that the clustering criteria we use (<2% divergence, including insertions and deletions) are more conservative than commonly used definitions of ‘species-level’ OTUs (<2% divergence excluding indels). We used conservative clustering because no universal cutoff applies to all organisms [51] and in order to avoid merging together organisms with potentially different phenotypes (for example, closely-related strains, see Additional file 3: Figure S4 for an example in closely-related *Escherichia/Shigella* OTUs). Similar considerations have led to the development of specialized software for the analysis of vaginal 16S rRNA gene survey data [52]. Our approach provides a good tradeoff between mitigating the effect of errors and allowing an unbiased analysis of the data. Furthermore, an exploration of increasingly permissive clustering thresholds reveals that our conservative clustering strategy does not lose statistical power (see Additional file 3: Figures S5, S6).

Chimera checking was performed with Uchime 4.2.40 [53].

### PICRUST analysis

The most abundant 6879 OTUs were reprocessed using QIIME [7] version 1.8.0-dev as recommended on the PICRUST website (specifically OTUs were constructed with the pick_closed_reference_otus.py script against the latest version (version 13.5) of the Greengenes [54] database) and the resulting information was processed with PICRUST [16] version 1.0.0-dev using the KEGG analysis module and aggregating the results to level 3. The results were further explored with STAMP [17] version 2.0.2, using the two-group analysis module, focusing on known aerobic and anaerobic pathways.

### Data normalization

In order to avoid the bias that may be introduced by preferential amplification or sequencing of specific sequences, we scaled the counts by the 56^th^ percentile of the number of OTUs in each sample. The 56^th^ percentile was empirically determined from the distribution of non-zero counts required to behave consistently across our samples. We normalized with a Cumulative Sum Scaling approach, which scales counts by dividing the sum of each sample’s counts up to and including the *p*th quantile (that is, for all samples *j*, *S*_*p*_ = ∑ _*i*_(*c*_*ij*_|*c*_*ij*_) ≤ *q*_*pj*_, where *q*_*pj*_ is the *p*^th^ quantile of sample *j*). Normalized counts are then given by $\frac{c_{\mathrm{ij}}}{S_{\mathrm{pj}}}1000$. This method constrains communities with respect to a total size, but does not place undue influence on features (OTUs) that are preferentially sampled. A full description of the methodology is provided in Paulson et al. [18].

### Statistical approaches

To test for presence and absence of an organism we performed Fisher’s test stratifying by positive and negative samples. Samples were stratified as positive for an organism if the sample had one or more sequences of the organism with a sample being negative if there was absence of sequences. The totals were calculated for each taxa, a minimum of 20 positive samples was required for a statistical test to be attempted. To correct for multiple comparisons we minimized the expected proportion of false positives following Benjamini and Hochberg [55].

Differential abundance was assessed with the package metagenomeSeq [18] - a statistical approach that models confounding such as age and country, and also the effect of undersampling on the observed counts. Significant findings were reported for OTUs that satisfied the following criteria: (1) OTU was abundant (≥12 normalized counts per sample) in cases or controls; (2) OTU was prevalent (present in ≥10 cases and controls); (3) OTU had fold change or odds ratio exceeding 2 in either cases or controls; and (4) statistical association was significant (*P* <0.05) after Benjamini-Hochberg correction for multiple testing.

Analyses were performed using the R software package 3.0.1 and packages, Vegan 2.0-7 and metagenomeSeq 1.2.21.

### Correlation network construction

Correlation networks were constructed separately on cases and controls to characterize the dependencies between 268 differentially abundant OTUs (Additional file 4: Table S3).

Each network was built using SparCC [56], a tool specifically developed for assessing the correlation structure within microbial communities. The statistical significance for each OTU-OTU-interaction was obtained with an empirical null distribution using 1,000 bootstrap iterations. The *P* values were further adjusted for multiple comparisons using the Benjamini and Hochberg [55] correction. All OTU-OTU-interactions with FDR < =0.05, were considered significant and were represented as edges in the network.

For simplicity of visual representation, OTUs were aggregated at genus or lower taxonomic levels using the median normalized abundance of the aggregated OTUs as the abundance of the corresponding taxonomic group. We omitted all taxonomic groups with median abundance lower than 500 normalized counts, as well as all edges with SparCC correlation lower than 0.09. The plots were drawn in Cytoscape 3.0.1 [57].

## Competing interests

The authors declare that they have no competing interests.

## Authors’ contributions

Sample collection and data management: MP, BRL, MA, MAH, JO, BT, MML, SP, KK, UNI, CE, MA, DA, FA, MTA, RA, SS, JBO, EO, JJ, EU, RO. 16S rRNA gene data collection and analysis: MP, AWW, JNP, BRL, VM, IA, HCB, RR, MDS, VM, JP, JPN, OCS. Study design: MP, AWW, MA, MAH, JO, VM, MML, RFB, JGM, DS, JP, OCS, JPN. Statistical analysis: MP, AWW, JNP, BL, IA, HCB, OCS. Writing: MP, AWW, JNP, BL, JPN, OCS. All authors read and approved the final manuscript.

## Supplementary Material

Additional file 1: Table S1 Proportional abundance of dominant bacterial genera in cases and controls, both overall and stratified by age stratum and country. Click here for file

Additional file 2: Table S2 Comparison of Shannon diversity across ages and countries. P-values computed with Tukey’s honestly significant difference test to account for multiple comparisons. Click here for file

Additional file 3 Additional figures (S1-S8) and figure captions. Click here for file

Additional file 4: Table S3 OTUs found to be significantly associated with diarrhea or with diarrhea free controls. Click here for file

Additional file 5: Table S4 Mapping of taxonomic names used in our paper and nearest hits to the corresponding 16S rRNA sequence. Due to the poor resolution of the 16S rRNA region used in our study we manually assigned each OTU to the most precise taxonomic level possible. In some cases a same organism appears in multiple groups, reflecting errors in the underlying database used (RDP version 10.4). For brevity, only ambiguous taxonomic groups are listed. Click here for file

Additional file 6: Table S5 Mapping of barcode information to sample IDs. Click here for file
