# Supplementary material for: Diarrhea in young children from low-income countries leads to large-scale alterations in intestinal microbiota composition
Source: Genome Biol. 2014 Jun 27;15(6):R76. doi: 10.1186/gb-2014-15-6-r76 (PMC4072981; doi:10.1186/gb-2014-15-6-r76)
Supplement: Additional file 3 — Additional figures (S1-S8) and figure captions. [file gb-2014-15-6-r76-S3.docx]

***Supplementary figures***

*Figure S1:* ***Shannon diversity indices across age and country.*** *Points represent the average Shannon diversity stratified by country, age, and health status. Each panel consists of data from a different country and colors represent the health status where red represents cases and green, controls. The upper and lower bars correspond to the 95% confidence interval. There is a strong trend for both cases and controls to become more complex with age. Diversity appears to increase greatly following the 6-11 month age group in Mali and The Gambia. Diversity increases throughout age in Kenya and Bangladesh.*

*
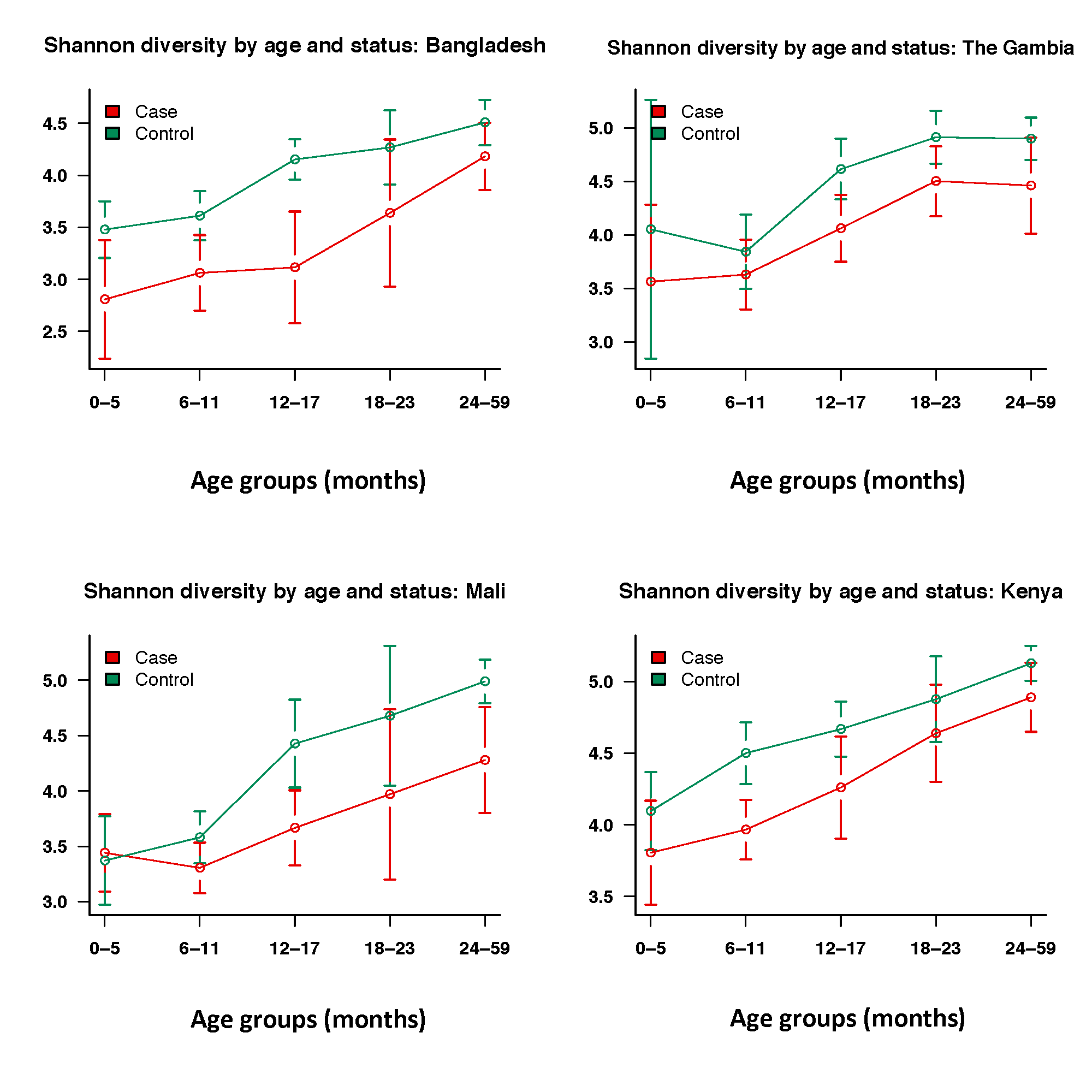
*

*Figure S2:* ***Proportional abundance of Prevotella, Escherichia/Shigella, Veillonella, and Streptococcus in non-diarrheal controls and MSD cases in different age categories.*** *The upper whisker extends from the 75th percentile to the highest value that is within 1.5 * IQR of the hinge, where IQR is the inter-quartile range, or distance between the first and third quartiles. The lower whisker extends from the hinge to the lowest value within 1.5 * IQR of the hinge. Data beyond the end of the whiskers are outliers and are not plotted. Each color represents a different age group. A bracket above the age groups indicates a statistically significant (p<0.01, linear regression) association between age and abundance of that genera.*

*
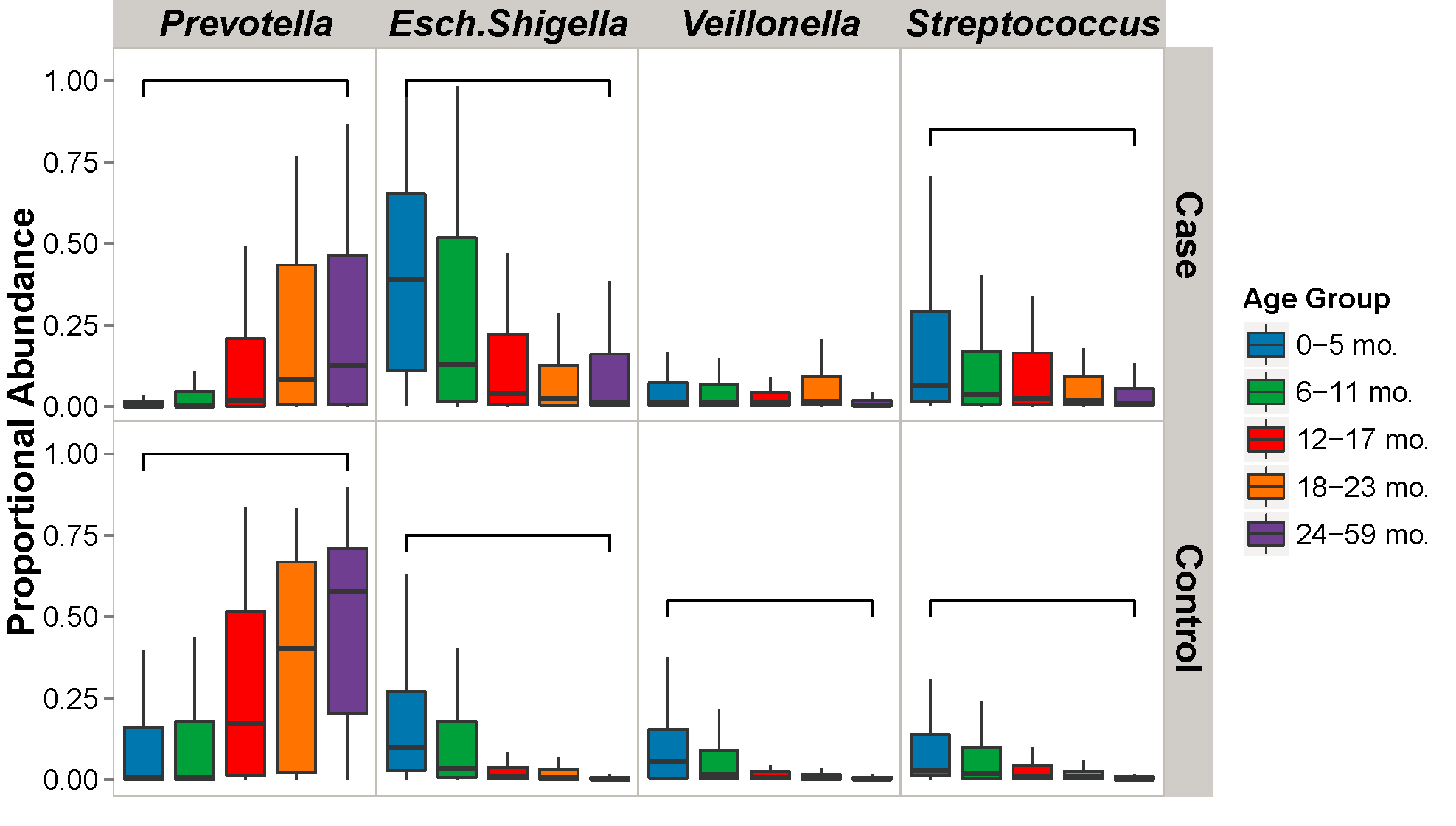
*

*Figure S3:* ***PCA analysis fails to distinguish cases from controls even after stratifying by age and country, likely due to high inter-personal variability.*** *The plots represent the first two components from a principal component analysis of the OTUs found to be most strongly associated with MSD. The distance used was Euclidean, however other commonly used distances also yield similar plots (data not shown). The columns correspond to country in the order: Gambia, Mali, Kenya and Bangladesh. The rows correspond to age categories from youngest to oldest. Controls are colored green and cases are colored orange.
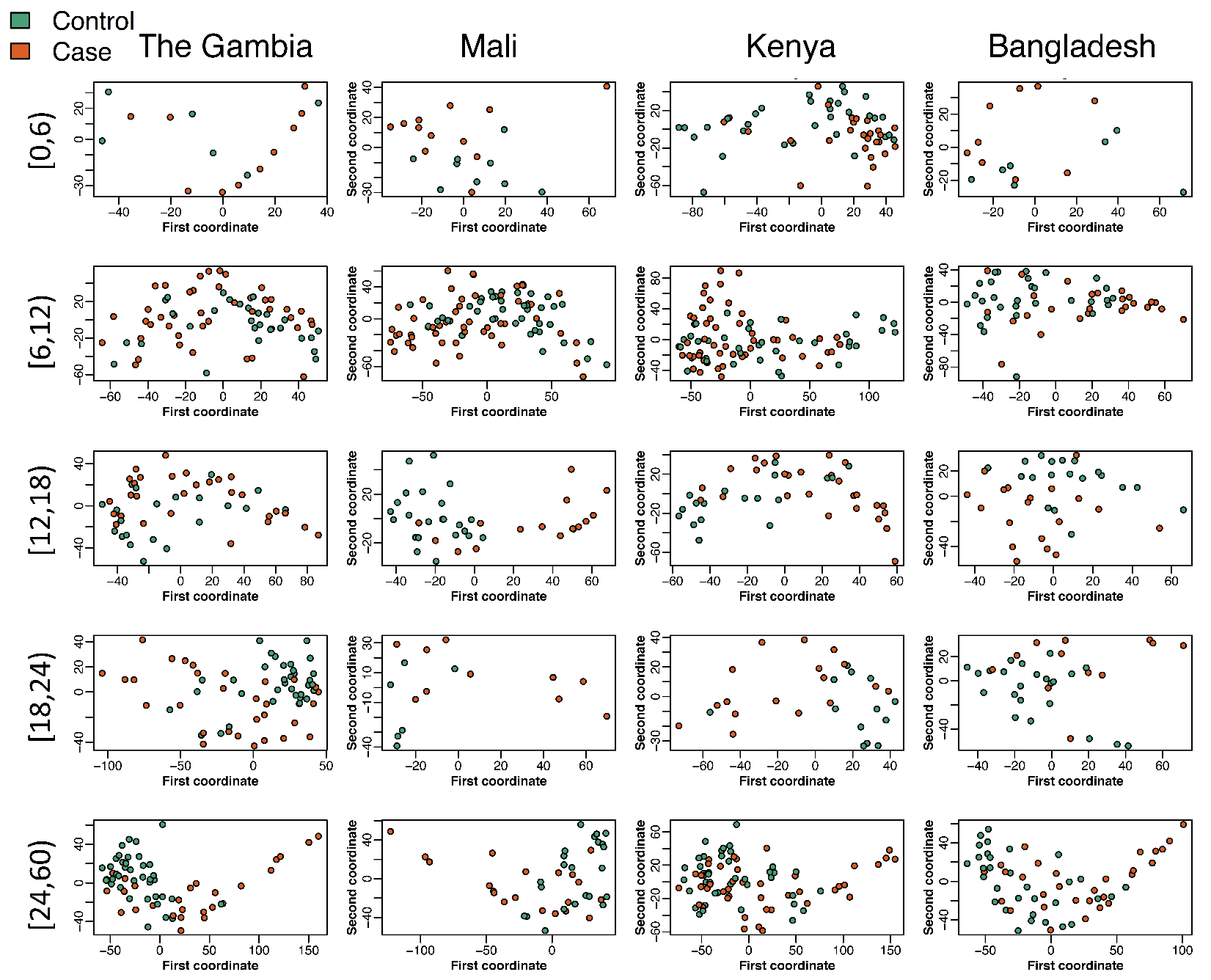
*

*Figure S4:* ***Loose clustering criteria may aggregate phenotypically distinct organisms.***  *Correlation between the abundance profiles of 19 different OTUs classified as Escherichia/Shigella. Despite high sequence similarity between the corresponding sequences, the individual OTUs (defined as DNAclust radius 0.01) have different abundance profiles, falling into roughly 6 distinct clusters, implying distributional or phenotypic differences between the corresponding organisms. The colored circles on the right represent the result of clustering the data at a looser radius of 0.02 (each cluster labeled by a different color). The phenotypically-defined and sequence-defined clusters are not fully concordant highlighting that sequence alone is not sufficient to define OTUs that are biologically relevant.*

*
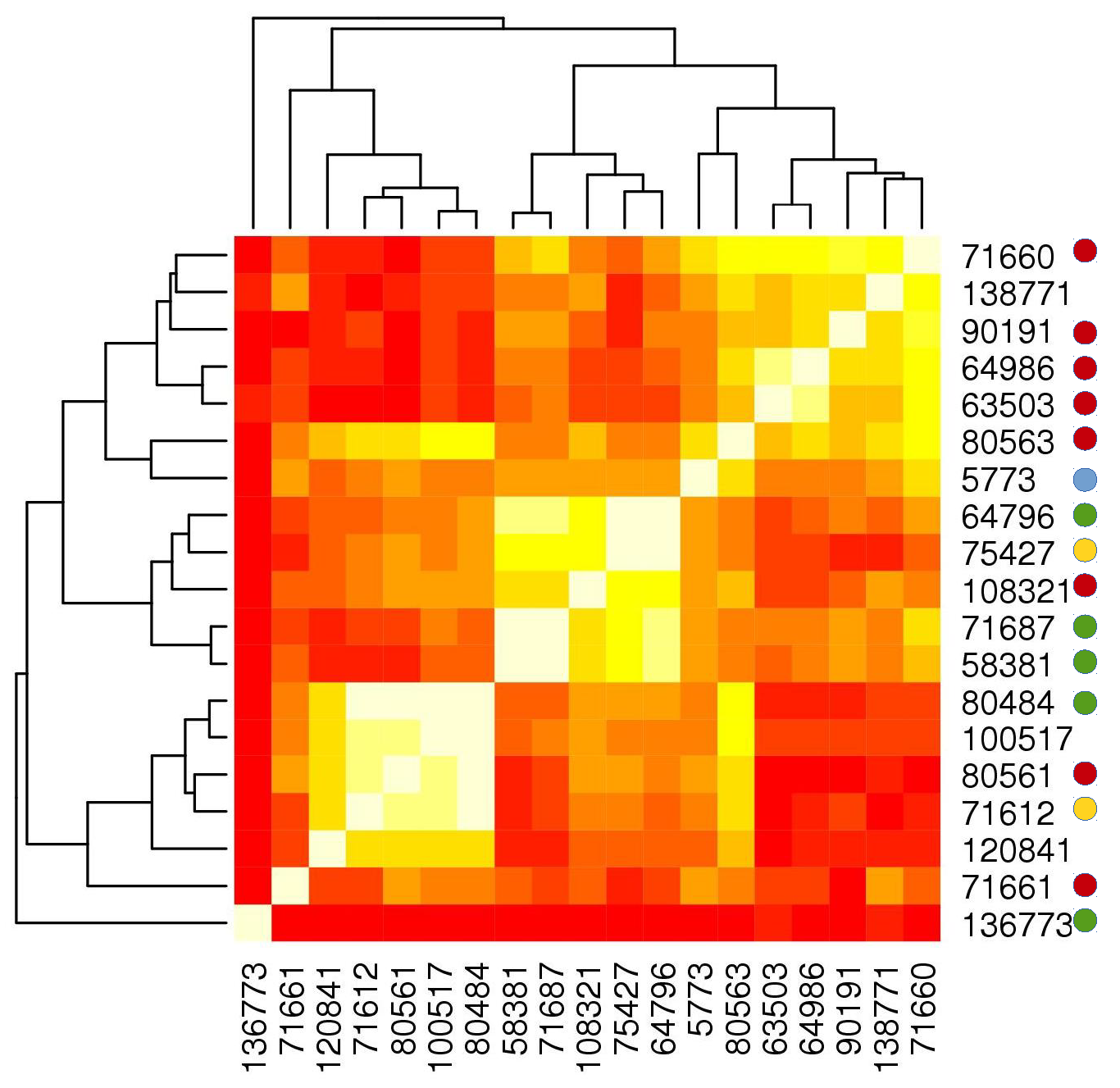
*

*Figure S5:* ***Differential abundance analysis is robust to changes in OTU radius.*** *(A) Approximate number of OTUs (see below) detected as sequence similarity cutoff is relaxed. As expected, the number of OTUs decreases with decreasing similarity stabilizing at approximately 95% similarity. (B) Relative difference between fold-changes (with respect to 98% identity) as sequence similarity cutoff is relaxed. On average, there is little effect to fold-change with an increase in radius size while there are increased number of outlier features (with bigger fold-change differences starting at 95% similarity). Approximate OTUs (A) were constructed from the set OTUs used in the main manuscript by re-clustering the representative reads for each OTU were with 97-90 % identity and log2 fold-changes for new OTU centers were tracked and compared to the original centers. Relative fold-change difference (B) of re-clustered OTUs is computed as* $\frac{|{fc}_{98}-{fc}_{xx}|}{{fc}_{98}}$*. Using 99% is necessary when attempting to distinguish species or even strains in a clinical dataset.*

*
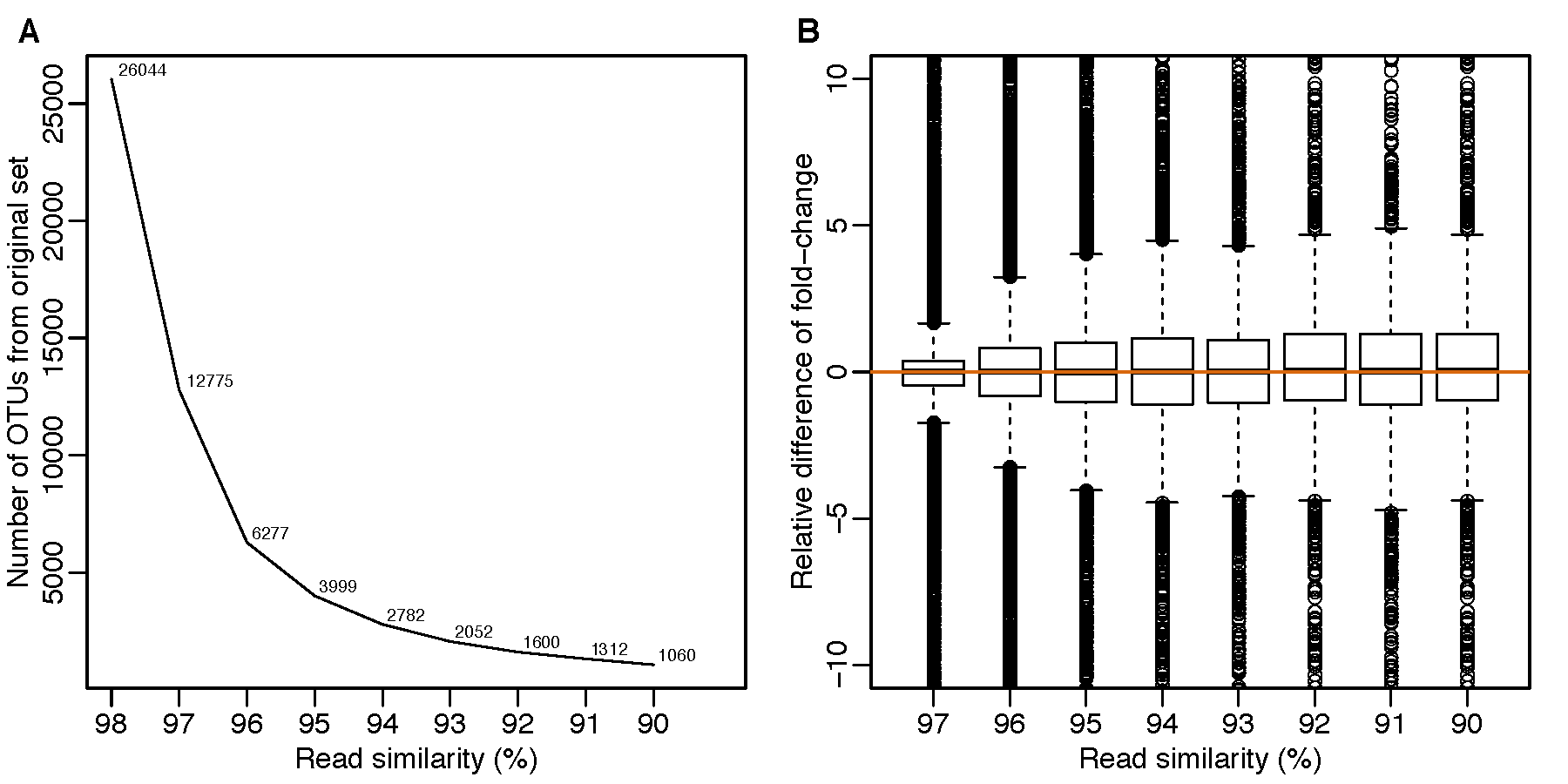
*

*Figure S6:* ***OTU prevalence is robust to OTU radius choice.*** *(****A and B****). Bar plot of the number of unique samples positive for Campylobacter jejuni (A) and Veillonella ratti (B) as OTU clusters annotated to each species are merged in order from most to least prevalent. For Campylobacter jejuni (A), known to be well-defined at the species level by 16S rRNA, prevalence at the OTU level (for the most prevalent cluster) is very close to prevalence at the species level. For Veillonella ratti, this ratio is smaller. (C) Ratio of OTU-level prevalence (for most prevalent OTU) to species-level prevalence for all species detected. The x-axis represents the log_2_ prevalence (number of positive samples) for the most prevalent OTU cluster in each annotated species in our dataset. The y-axis is the log_2_ ratio between the total prevalence of a species (sum of all OTUs) and the prevalence of the most abundant OTU. Campyllobacter jejuni (A) and Veillonella ratti (B) are shown as green points. Red points are those features in the top percentile (with largest ratio) where the species-level prevalence increase by at least 4.09 times. The size of each point corresponds to the number of OTUs with the same species-level annotation. For 82% of species this ratio is below 1, i.e., aggregating over all OTUs labeled with the same species label would at most double the prevalence of a specific species. This result is consistent with the observation that, for the majority of species, prevalence at the OTU level captures species level prevalence as well. The extreme outliers are 8 organisms (red dots) classified as Enterobacter sp. Y5, Prevotella intermedia, Prevotella sp. Oral clone BP1-16, Ruminococcus albus, Salmonella bongori, Shigella boydii, Streptococcus dysgalactiae, and Veillonella sp. MY-P9.*

**

*Figure S7.* ***Correlation networks constructed on the controls (A) and on the MSD case (B) samples.*** *Each node represents a taxon, and each edge represents the presence of significant correlations between OTUs assigned to the corresponding taxa. The diameter of a node is proportional to the normalized abundance of the particular taxon. The edge width is proportional to the number of OTU-OTU correlations linking together the corresponding taxa. Negative correlations are represented by red edges and positive correlations are represented by green edges. For clarity, only dominant genera and correlations shown (genera with > 500 normalized abundance, median SPARCC [17] correlation > 0.09). Broad taxonomic groups are highlighted through shaded areas. The broken line bisects the figure into obligate anaerobic lineages (left hand side) and facultatively anaerobic/microaerophilic lineages (right hand side).*

*
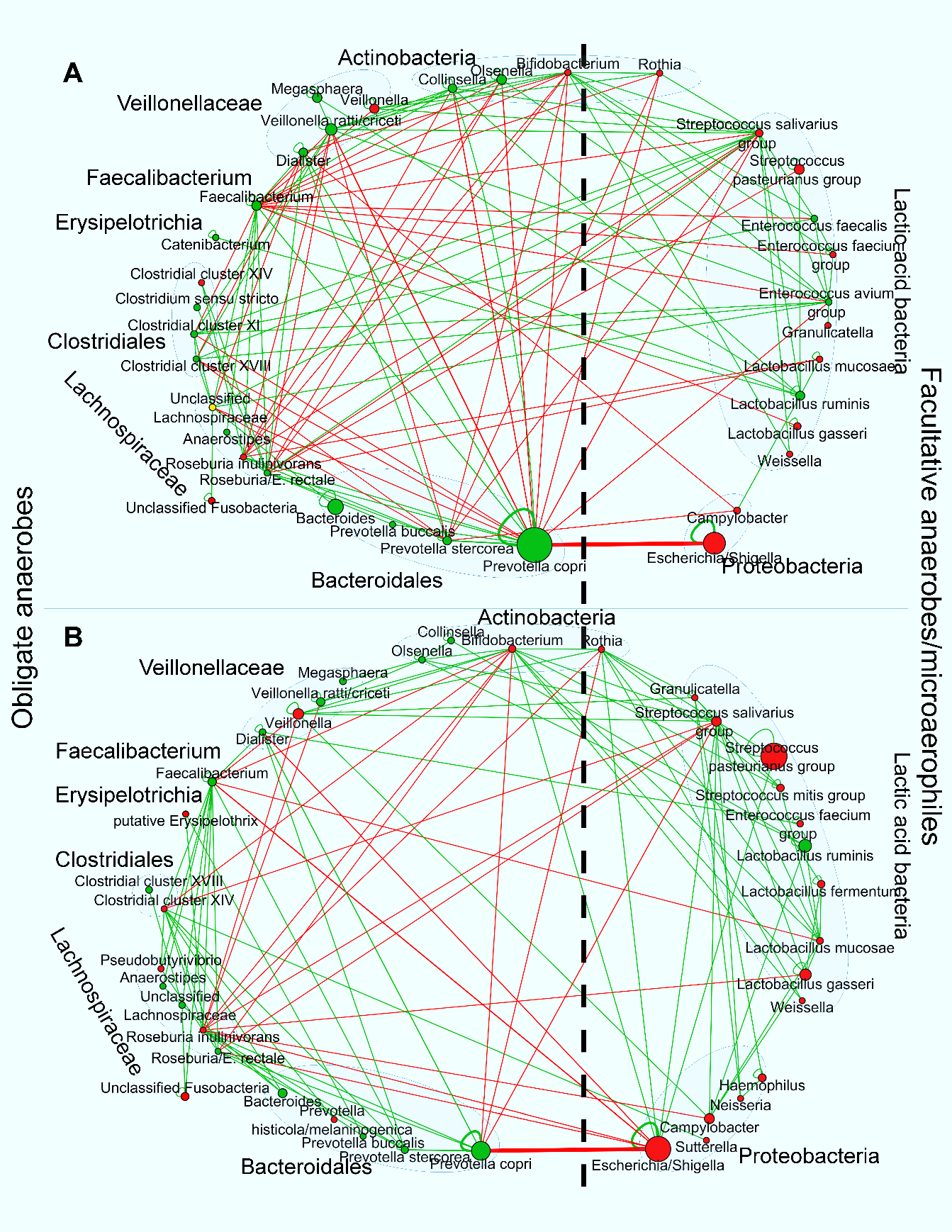
*
