# Supplementary material for: Diarrhea in young children from low-income countries leads to large-scale alterations in intestinal microbiota composition
Source: Genome Biol. 2014 Jun 27;15(6):R76. doi: 10.1186/gb-2014-15-6-r76 (PMC4072981; doi:10.1186/gb-2014-15-6-r76)
Supplement: Additional file 5: Table S4 — Mapping of taxonomic names used in our paper and nearest hits to the corresponding 16S rRNA sequence. Due to the poor resolution of the 16S rRNA region used in our study we manually assigned each OTU to the most precise taxonomic level possible. In some cases a same organism appears in multiple groups, reflecting errors in the underlying database used (RDP version 10.4). For brevity, only ambiguous taxonomic groups are listed. [file gb-2014-15-6-r76-S5.docx]

*Table S3:* ***Mapping of taxonomic names used in our paper and nearest hits to the corresponding 16S rRNA sequence.*** *Due to the poor resolution of the 16S rRNA region used in our study we manually assigned each OTU to the most precise taxonomic level possible. In some cases a same organism appears in multiple groups, reflecting errors in the underlying database used (RDP version 10.4). For brevity, only ambiguous taxonomic groups are listed.*

|  | **Mapping of taxonomic names to database nearest hits** |
| --- | --- |
|  |  |
| **Taxonomic label** | **Species/genera aggregated** |
| *Haemophilus* | *Haemophilus, Actinobacillus, Terrahaemophilus* |
| *Streptococcus pasteurianus* group | *S. pasteurianus, S. gallolyticus, S. macedonicus, S. lutetiensis, S. infantarius, S. equinus, S. luteciae* |
| *Streptococcus mitis* group | *S. mitis, S. pneumoniae, S. oralis, S. peroris*, *S. pseudopneumoniae, S. australis, S. infantis* |
| *Streptococcus salivarius* group | *S. salivarius, S. vestibularius, S. thermophilus* |
| *Veillonella* | *V. atypica, V. rogosae, V. caviae, V. parvula, V. denticariosi* |
| *Lactobacillus gasseri* | *L. gasseri, L. johnsonii, L. taiwanensis* |
| *Escherichia/Shigella* | *Enterobacter* spp., *Citrobacter* spp., *Shigella* spp., *Escherichia* spp. |
| *Klebsiella* | *Klebsiella* spp., *Cronobacter* spp., *Raoultella* spp., *Enterobacter* spp. |
| *Bacteroides* | *B. fragilis, B. ovatus, B. thetaiotaomicron, B. dorei, B. vulgatus* |
| *Blautia* | *B. wexlerae, B. luti* |
| *Campylobacter* | *C. upsaliensis, C. helveticus, C. jejuni, C. coli, C. insulaenigrae, C. peloridis, C. consisus* |
| *Clostridial cluster XI* | *C. difficile, C. bartletti, C. glycolicum* |
| *Clostridial cluster XIV* | *C. lactatifermitans* |
| *Clostridial cluster XV* | *C. callanderi, C. limosum* |
| *Clostridial cluster XVI* | *C. innocuum* |
| *Clostridial cluster XVIII* | *C. ramosum* |
| *Clostridium sensu stricto* | *C. tertium, C. sartagoforme, C. carnis, C. chauvoei, C. septicum, C. gasigenes, C. paraputrificum, C. saccharobutylicum, C. butyricum, C. disporicum* |
| *Dorea* | *D. formicigenerans, D. xylanolyticum, D. algidixylanolyticum, D. saccharolyticum, D. longicatena* |
| *Enterococcus avium group* | *E. avium, E. gilvus, E. raffinosus, E. devriesei, E. pseudoavium, E. malodoratus, E. mundtii, E. thailandicus, E. hirae, E. durans* |
| *Enterococcus faecium group* | *E. faecium* |
| *Fusobacterium* | *F. nucleatum, F. periodonticum, F. canifelinum, F. alocis* |
| *Gemella* | *G. haemolysans, G. sanguinis, G. morbillorum* |
| *Granulicatella* | *G. adiacens, G. elegans* |
| *Leuconostoc* | *L. lactis, L. holzapfelii, L. carnosum, L. kimchii, L. mesenteroides, L. citreum, L. gasicomitatum* |
| *Mitsuokella/Selenomonas* | *M. multiacida, M. jalaludinii, S. ruminantium* |
| *Neisseria* | *N. subflava, N. flavescens, N. animalis, N. elongata, N. polysaccharea* |
| *Prevotella histicola/melaninogenica* | *P. histicola, P. melaninogenica* |
